# Complete chloroplast genome sequence of *Betula platyphylla*: gene organization, RNA editing, and comparative and phylogenetic analyses

**DOI:** 10.1186/s12864-018-5346-x

**Published:** 2018-12-20

**Authors:** Sui Wang, Chuanping Yang, Xiyang Zhao, Su Chen, Guan-Zheng Qu

**Affiliations:** State Key Laboratory of Tree Genetics and Breeding, Northeast Forestry University, 26 Hexing Road, Harbin, 150040 China

**Keywords:** *Betula platyphylla*, White birch, Chloroplast genome, RNA editing, Phylogeny

## Abstract

**Background:**

*Betula platyphylla* is a common tree species in northern China that has high economic and medicinal value. Our laboratory has been devoted to genome research on *B. platyphylla* for approximately 10 years. As primary organelle genomes, the complete genome sequences of chloroplasts are important to study the divergence of species, RNA editing and phylogeny. In this study, we sequenced and analyzed the complete chloroplast (cp) genome sequence of *B. platyphylla*.

**Results:**

The complete cp genome of *B. platyphylla* was 160,518 bp in length, which included a pair of inverted repeats (IRs) of 26,056 bp that separated a large single copy (LSC) region of 89,397 bp and a small single copy (SSC) region of 19,009 bp. The annotation contained a total of 129 genes, including 84 protein-coding genes, 37 tRNA genes and 8 rRNA genes. There were 3 genes using alternative initiation codons. Comparative genomics showed that the sequence of the Fagales species cp genome was relatively conserved, but there were still some high variation regions that could be used as molecular markers. The IR expansion event of *B. platyphylla* resulted in larger cp genomes and *rps19* pseudogene formation. The simple sequence repeat (SSR) analysis showed that there were 105 SSRs in the cp genome of *B. platyphylla*. RNA editing sites recognition indicated that at least 80 RNA editing events occurred in the cp genome. Most of the substitutions were C to U, while a small proportion of them were not. In particular, three editing loci on the rRNA were converted to more than two other bases that had never been reported. For synonymous conversion, most of them increased the relative synonymous codon usage (RSCU) value of the codons. The phylogenetic analysis suggested that *B. platyphylla* had a closer evolutionary relationship with *B. pendula* than *B. nana*.

**Conclusions:**

In this study, we not only obtained and annotated the complete cp genome sequence of *B. platyphylla*, but we also identified new RNA editing sites and predicted the phylogenetic relationships among Fagales species. These findings will facilitate genomic, genetic engineering and phylogenetic studies of this important species.

**Electronic supplementary material:**

The online version of this article (10.1186/s12864-018-5346-x) contains supplementary material, which is available to authorized users.

## Background

*Betula platyphylla*, or Asian white birch, is a broad-leaved deciduous hardwood tree species that belong to the genus *Betula*, in the family Betulaceae. It is a pioneer tree species that can rapidly colonize open ground, especially in secondary successional sequences. It grows in the temperate or subarctic regions of Asia, including Japan, China, Korea, and Siberia. The grayish bark of this tree is marked with long, horizontal lenticels that often separates into thin, papery plates, which is the most typical characteristic of this tree species [1, 2]. *B. platyphylla* is often used as a wayside tree or landscape tree species because of its graceful shape. It is a valuable commercial tree species that is harvested for lumber and pulpwood for paper production [3]. Recent studies have indicated that birch bark contains numerous triterpenoids and has substantial medicinal value [4, 5].

As primary plastids found only in plant cells and eukaryotic algae, chloroplasts are semiautonomous organelles that are not only perform photosynthesis but also participate in a range of biochemical processes. Chloroplasts are believed to have arisen from an endosymbiotic event and have their own genomes, which are often abbreviated as cp or ct [6, 7]. Since the first cp genomes were sequenced in 1986, more than 2500 complete cp genome sequences have been released in the National Center for Biotechnology Information (NCBI) organelle genome database as of March 2018 [8, 9]. The advent of next-generation-sequencing (NGS) technologies has facilitated rapid progress in the field of cp genomics [10]. In the future, due to the popularity of third-generation sequencing, longer average read lengths will make it easier to assemble cp genomes [11–13]. For most land plants, cp genomes have highly conserved structures and are circular DNA molecules that comprise two inverted repeats (IR), which separate a large and a small single copy (LSC and SSC) region. Chloroplast genome sizes vary between species, ranging from 107 kb (*Cathaya argyrophylla*) to 218 kb (*Pelargonium × hortorum*), with an average size of approximately 150 kb [14, 15]. There are approximately 120–130 genes in the cp genome, which participate primarily in photosynthesis, transcription, and translation [16]. RNA editing, which is a posttranscriptional modification phenomenon, occurs in some transcripts of these cp genes. Editing by insertion, deletion or switching bases, such as cytidine (C) to uridine (U), is an essential repair mechanism, and many mutations at the cp genome level may lead to strong deleterious phenotypes [17]. Because they have fairly stable structures, moderate evolutionary rates and uniparental inheritance in most angiosperms, the cp genomes have made significant contributions to phylogenetic studies [16, 18].

In this study, we aimed to determine the complete cp genome sequence of *B. platyphylla* and to characterize its genome structure, gene content and other characteristics. Furthermore, we recognized RNA editing sites in the whole cp genome of *B. platyphylla* using RNA-Seq data. We predicted their relationships through a comparative analysis with other Fagales species cp sequences within phylogenetic clades.

## Materials and methods

### Plant materials and sequencing

Tender leaves were collected from an adult *B. platyphylla* plus tree that is located on the Northeast Forestry University campus. Total genomic DNA was extracted from tender leaves using the CTAB method [19]. Three paired-end (insert sizes = 200 bp, 500 bp and 800 bp) and three mate-pair (insert sizes = 2 kbp, 5 kbp and 10 kbp) Illumina libraries were prepared and sequenced on the HiSeq 2000 platform (Illumina, USA) at BGI (Shenzhen,Guangdong,China).

### Data filtration and cp DNA sequence extraction

To obtain high-quality and vector/adaptor-free reads, raw paired-end reads were filtered using the NGSQC Toolkit v2.3.3 (cut-off read length for HQ = 70%, cut-off quality score = 20, trim reads from 5′ = 3, trim reads from 3′ = 7) [20]. The qualities of the clean reads were checked using FastQC (v0.11.5). To identify the cp sequences, all of the clean reads, which included sequences from both the nucleus and organelles, were mapped to the complete cp genome sequences of 2670 plant species, which were downloaded from the NCBI Organelle Genome Resources database (www.ncbi.nlm.nih.gov/genome/organelle/) using BWA (v0.7.13) [21]. Finally, we extracted cp sequences from the SAM files and obtained three files of paired-end reads.

### Genome assembly and annotation

For de novo cp genome assembly, an Edena assembler (v3.131028) with default parameters was used to assemble all the paired-end sequences into contigs [22]. Next, neighboring contigs with paired-end or mate-pair support for continuity were merged into scaffolds using SSPACE (v3.0) [23]. Then, using the cp genome sequences of two other reference Fagales plants, *Betula nana* (KX703002.1) and *Ostrya rehderiana* (KT454094.1), a single cp sequence with gaps was assembled. After that, GapCloser (v1.12) was used to close most of the gaps, and Sanger sequencing was used to fill residual gaps. The complete cp genome sequence was further checked using BWA.

Except for tRNA genes, which were verified using tRNAscan-SE 2.0, the *B. platyphylla* cp genome sequence was annotated using the online Chloroplast Genome Annotation, Visualization, Analysis and GenBank Submission Tool (CpGAVAS) [24, 25]. First, AnnotateGenome was utilized to obtain the primitive annotation results in the GFF3 format. Second, we used AnnotateGene and Apollo Genome Annotation and Curation Tool (v1.11.8) to manually correct the abnormal features based on the reference database of CpGAVAS and the tRNA genes annotated by tRNAscan-SE. Last, OrganellarGenomeDRAW was used to directly generate a corrected cp circular map [26].

### Codon usage and alternative start codons statistics

Codon usage was determined for all protein-coding genes (RNA sequences without editing). To examine the deviation in synonymous codon usage while avoiding the influence of the amino acid composition, the relative synonymous codon usage (RSCU) was calculated with MEGA 7 software (version 7.0.18).

Three cp genes (*rps19*, *psbC* and *ndhD*) were annotated with the Non-ATG start codon in the *B. platyphylla* cp genome, we selected these genes from 30 model plant and representative plant species according to the Angiosperm Phylogeny Group (APG) IV system (Additional file 1: Table S1). Then, the sequence logos of the first 10 bp of the three genes across the species were created using the WebLogo 3 application (http://weblogo.threeplusone.com/). We also visualized the RNA-Seq mapping of these sites and aligned them with the sequence logos.

### Genome comparison

The complete cp genome sequences of *B. platyphylla* and four other closely related species, *B. pendula* (LT855378.1), *B. nana* (KX703002.1), *Corylus chinensis* (KX814336.2) of Betulaceae and *Juglans sigillata* (KX424843.1) of Juglandaceae, were compared using the program mVISTA. EMBOSS Stretcher, a modification of the Needleman-Wunsch algorithm that allows larger sequences to be aligned globally, was used to align these cp genome sequences to obtain accurate identity and similarity.

### IR expansion and contraction

Depending on the classification system for Fagales taxa, four species *Betula platyphylla*, *Juglans regia* (MF167463.1), *Morella rubra* (KY476637.1) and *Castanea mollissima* (KY951992.1) were selected to represent the families Betulaceae, Juglandaceae, Myricaceae and Fagaceae, respectively.

### SSR analysis

Simple sequence repeats (SSRs) were detected using the Perl script MISA (MIcroSAtellite identification tool) by setting the minimum number of repeats to 10, 5, 4, 3, 3 and 3 for mono-, di-, tri-, tetra-, penta- and hexanucleotides, respectively. Meanwhile, CandiSSR was used to identify polymorphic SSRs (PolySSRs) and to automatically design primer pairs for each identified PolySSR in the three *Betula* species [27].

### Recognition of RNA editing sites

In this study, an RNA-Seq experiment with 3 individual leaf samples was used to identify RNA editing events. The total RNA was extracted from mature foliage using an Extract kit (RP3301, BioTeke, China). The RNA-Seq library construction and sequencing were performed at Novogene Bioinformatics Technology Co., Ltd. (Beijing, China). The filtered paired-end reads obtained from an Illumina HiSeq 2000, were aligned to the *B. platyphylla* cp genome using HISAT2 (v2.1.0) software with strict comparison conditions. SAMtools (v1.9), bedtools (v2.25.0) and ChloroSeq were used to call and analyse precise RNA editing sites [28]. Because SNPs or mismatches may interfere with the results, we also mapped the set of PE 100 bp-long reads that was used to assemble the *B. platyphylla* cp genome back to the cp genome sequence using bowtie2 (v2.3.4.1) software and then checked the SNPs. Finally, we designed several pairs of primers using Primer Premier 6.0 software (PREMIER Biosoft International, Canada) and amplified the target sequence by PCR to form genomic DNA (gDNA) and complementary DNA (cDNA). The target representative editing sites were confirmed by Sanger sequencing. The relevant primer information is summarized in Additional file 1: Table S2.

### Phylogenetic analysis and character evolution

The whole cp genome sequences of 21 species of Fagales were used to build a phylogenetic tree to confirm the genetic relationship among closely related species of *B. platyphylla*. In this phylogenetic tree, *Nicotiana tabacum* was used as the out-group. Nucleotide sequences were aligned using MAFFT (version 7.294b). All alignments were checked and adjusted manually. The program MEGA-CC (version 7.0.26–1) was employed to find an optimal substitution model and to build a maximum likelihood (ML) phylogenetic tree. Bootstrap resampling with 500 replicates was used to evaluate the branch supports. More information is summarized in the Additional file 1: Table S3.

## Results

### Chloroplast genome assembly

Based on the NCBI Organelle Genome Resources database, we extracted approximately 128.8 Mbp of paired-end reads for cp genome assembly. With the help of Edena, a first assembly consisting of 35 contigs was obtained (Table 1). Further scaffolding with all of the paired-end and mate-pair reads resulted in a single scaffold under the guidance of the reference sequences. After using GapCloser to close most of the gaps, only two gaps remained. Finally, with the aid of Sanger sequencing, we filled the gaps, identified both ends of the sequence and obtained a circular cp genome.

**Table 1:** Statistics for the contigs

Number	Size (bp)	N50 (bp)	N90 (bp)	Longest length (bp)	Shortest length
35	150,362	21,011	2253	35,505	139

The whole cp genome of *B. platyphylla* had a length of 160,518 bp. Like most land plants, the circular cpDNA had typical quadripartite structures. An LSC region of 89,397 bp and an SSC region of 19,009 bp were separated by a pair of IR regions of 26,056 bp. The overall GC content of the *B. platyphylla* cp genome was 36.06%, and the GC contents of the LSC and the SSC regions were 33.66 and 29.76%, respectively. Because each IR region contained relatively abundant GC-rich rRNA and tRNA genes, the GC content of the IR region was 42.48%, which was much higher than that of the LSC and SSC regions.

### Chloroplast genome annotation

**Fig. 1:**
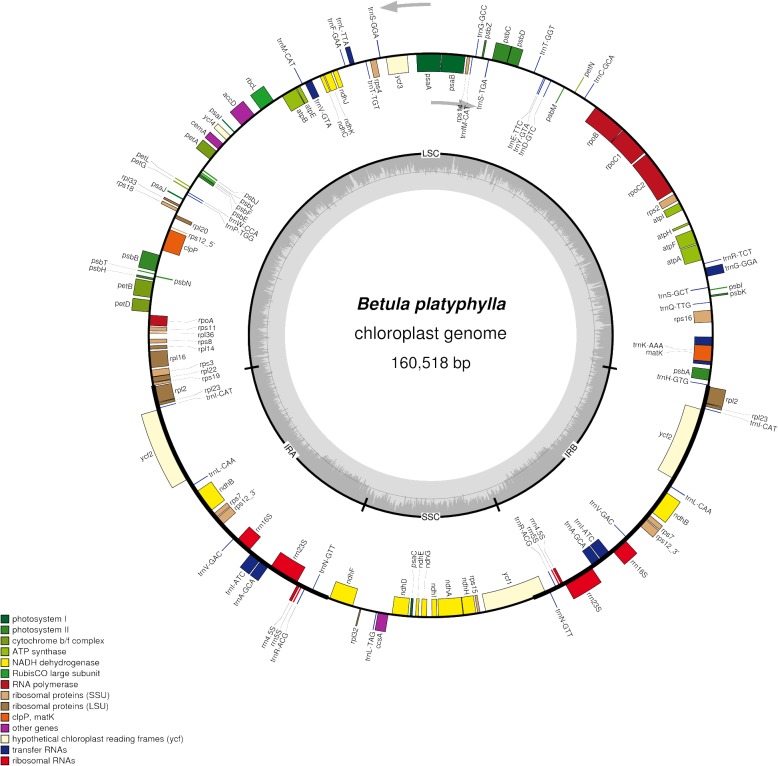
Chloroplast genome map of *Betula platyphylla*. As indicated by the arrows, genes inside the circle are transcribed clockwise, and genes outside are transcribed counter-clockwise. The grey inner circle corresponds to the GC content. Genes belonging to different functional groups are shown in different colours

A total of 129 genes were predicted to be encoded in the *B. platyphylla* cp genome, including 84 protein-coding genes, 37 tRNA genes and 8 rRNA genes. Among them, 95 genes were unique, and 17 genes were duplicated in the IR regions. By calculating the GC content of the genes, we found that it was higher in the rRNAs (54.89%) and tRNAs (53.20%) than in the protein-coding genes (36.93%). The majority of 112 unigenes were single-exon genes, while 18 genes (12 protein-coding genes and 6 tRNA genes) contained 2 exons and only 4 protein-coding genes contained 3 exons. All of the genome and annotation information is shown in Fig. 1.

Among the *B. platyphylla* cp genes, several were special. The *rps12* gene was a trans-spliced gene that consisted of 3 exons that code for the homologous ribosomal protein S12. C-terminal exons 2 and 3 of *rps12* were located in each IR region, but exon 1 was located in the LSC, approximately 28 kbp downstream of the nearest copy of exon 2, which was located in one of the IR regions and 61 kbp away from the other copy of exon 3, which was located in the other IR region. Prediction of the *B. platyphylla* cp gene function was based on homology, as these genes code for a variety of proteins, mostly involved in photosynthesis and other metabolic processes. Regarding photosynthesis, a subset of the genes synthesizes the large Rubisco subunit and thylakoid proteins. In addition, other genes encode subunits of a protein complex that mediates redox reactions to recycle electrons. Table 2 shows the gene functions and groups in the *B. platyphylla* cp genome.

**Table 2:** Group of genes within the *B. platyphylla* chloroplast genome

Group of genes	Gene names
Photosystem I	*psaA*, *psaB*, *psaC*, *psaI*, *psaJ*
Photosystem II	*psbA*, *psbB*, *psbC*, *psbD*, *psbE*, *psbF*, *psbH*, *psbI*, *psbJ*, *psbK*, *psbL*, *psbM*, *psbN*, *psbT*, *psbZ*
Cytochrome b/f complex	*petA*, *petB*, *petD*, *petG*, *petL*, *petN*
ATP synthase	*atpA*, *atpB*, *atpE*, *atpF*, *atpH*, *atpI*
NADP dehydrogenase	*ndhA*, *ndhB*, *ndhC*, *ndhD*, *ndhE*, *ndhF*, *ndhG*, *ndhH*, *ndhI*, *ndhJ*, *ndhK*
RubisCO large subunit	*rbcL*
RNA polymerase	*rpoA*, *rpoB*, *rpoC1*, *rpoC2*
Ribosomal proteins (SSU)	*rps2*, *rps3*, *rps4*, *rps7*, *rps8*, *rps11*, *rps12*, *rps14*, *rps15*, *rps16*, *rps18*, *rps19*
Ribosomal proteins (LSU)	*rpl2*, *rpl14*, *rpl16*, *rpl20*, *rpl22*, *rpl32*, *rpl33*, *rpl36*
Hypothetical chloroplast reading frames(ycf)	*ycf1*, *ycf2*, *ycf3*, *ycf4*
Other genes	*accD*, *ccsA*, *cemA*, *clpP*, *matK*
Ribosomal RNAs	*rrn4.5S*, *rrn5S*, *rrn16S*, *rrn23S*
Transfer RNAs	*trnA-GCA*, *trnC-GCA*, *trnD-GTC*, *trnE-TTC*, *trnF-GAA*, *trnfM-CAT*, *trnG-GCC*, *trnG-GGA*, *trnH-GTG*, *trnI-ATC*, *trnl-CAT*, *trnK-AAA*, *trnL-CAA*, *trnL-TAG*, *trnL-TTA*, *trnM-CAT*, *trnN-GTT*, *trnP-TGG*, *trnQ-TTG*, *trnR-ACG*, *trnS-GCT*, *trnS-GGA*, *trnS-TGA*, *trnT-GGT*, *trnT-TGT*, *trnV-GAC*, *trnV-GTA*, *trnW-CCA*, *trnY-GTA*

### Codon usage and alternative initiation codons statistics

It is generally acknowledged that codon biases reflect a balance between mutational biases and natural selection for translational optimization. We further analyzed the codon usage frequency and RSCU value in the *B. platyphylla* cp genome. It was not clear whether RNA editing occurred in these areas because there were some regions covered with no reads in our experiment, and the editing rates were not 100%. Here, we used RNA sequences without editing to compute the codon usage and RCSU values. We estimated that this would not have a large impact on the results. There were 84 protein-coding genes in the *B. platyphylla* cp genome, including 26,298 codons in total. Among the codons, the three amino acids present in the highest proportions were leucine (10.49%), isoleucine (8.97%) and serine (7.49%). Excluding the stop codons, cysteine (1.16%) was the least abundant amino acid (Additional file 1: Table S4, Figure S1). Codon usage was biased towards A and U at the third-codon position, which is similar to the trend that was observed in a majority of angiosperm cp genomes [29].

**Fig. 2:**
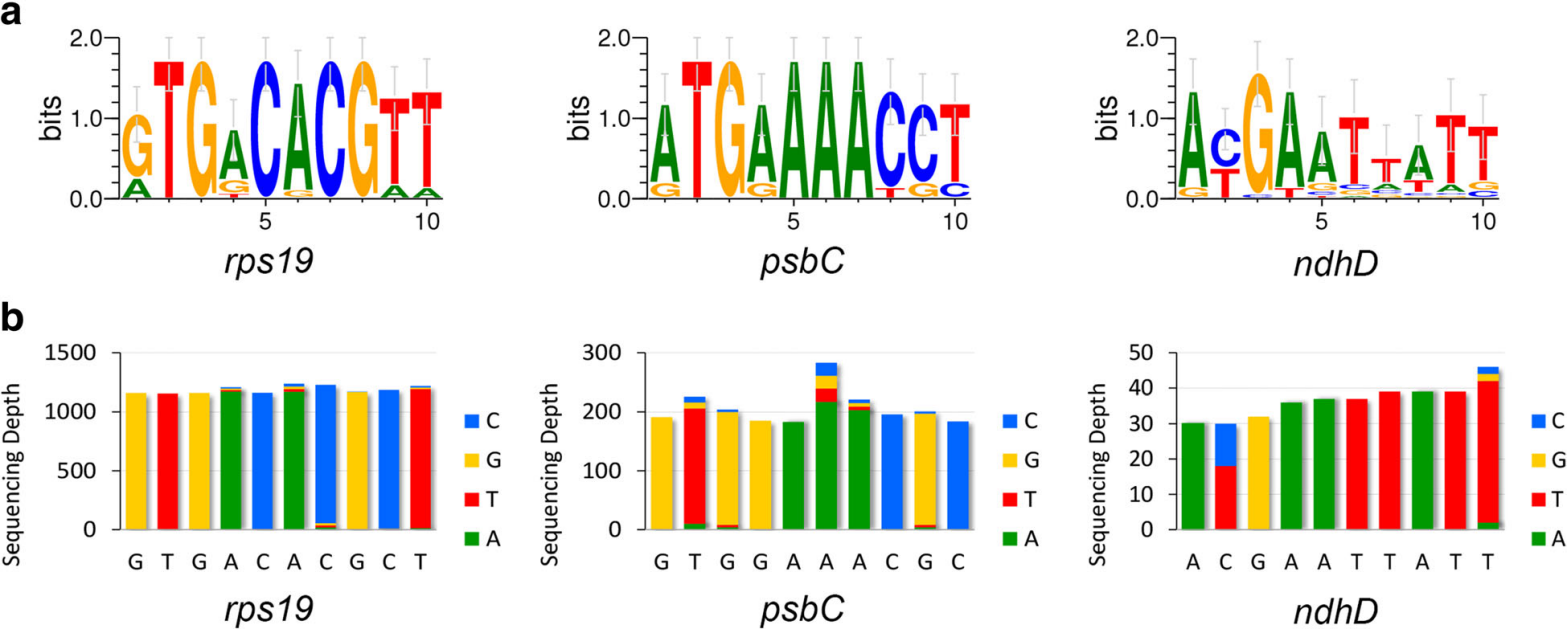
Sequence logo and RNA-Seq mapping of the three genes. **a**: Sequence logo of the first 10 bp of the three genes across the species. **b**: RNA-Seq mapping of the first 10 bp of the three genes in *B. platyphylla*

Unlike ordinary genes that use ATG as their initiation codons, several cp genes use other codons as exceptions. In the *B. platyphylla* cp genome, three genes were annotated with Non-ATG start codon: GTG was used by *rps19* and *psbC* and ACG was used by *ndhD*. These three genes are involved in translation, photosynthesis and respiration, respectively. As shown in Fig. 2a, these selected gene sites were relatively conserved across species. GTG was the dominant initiation codon in *rps19*, but not in *psbC*, and about half of the species took ACG as the start codon in *ndhD*. Figure 2b shows that, at the transcriptional level, the initiation codon of *rps19* and the *psbC* transcripts of *B. platyphylla* did not change significantly. However, editing ACG to AUG at the *ndhD* start codon was obvious and made its start codon go back to normal.

### Comparison of chloroplast genome sequences with those of other species

**Fig. 3:**
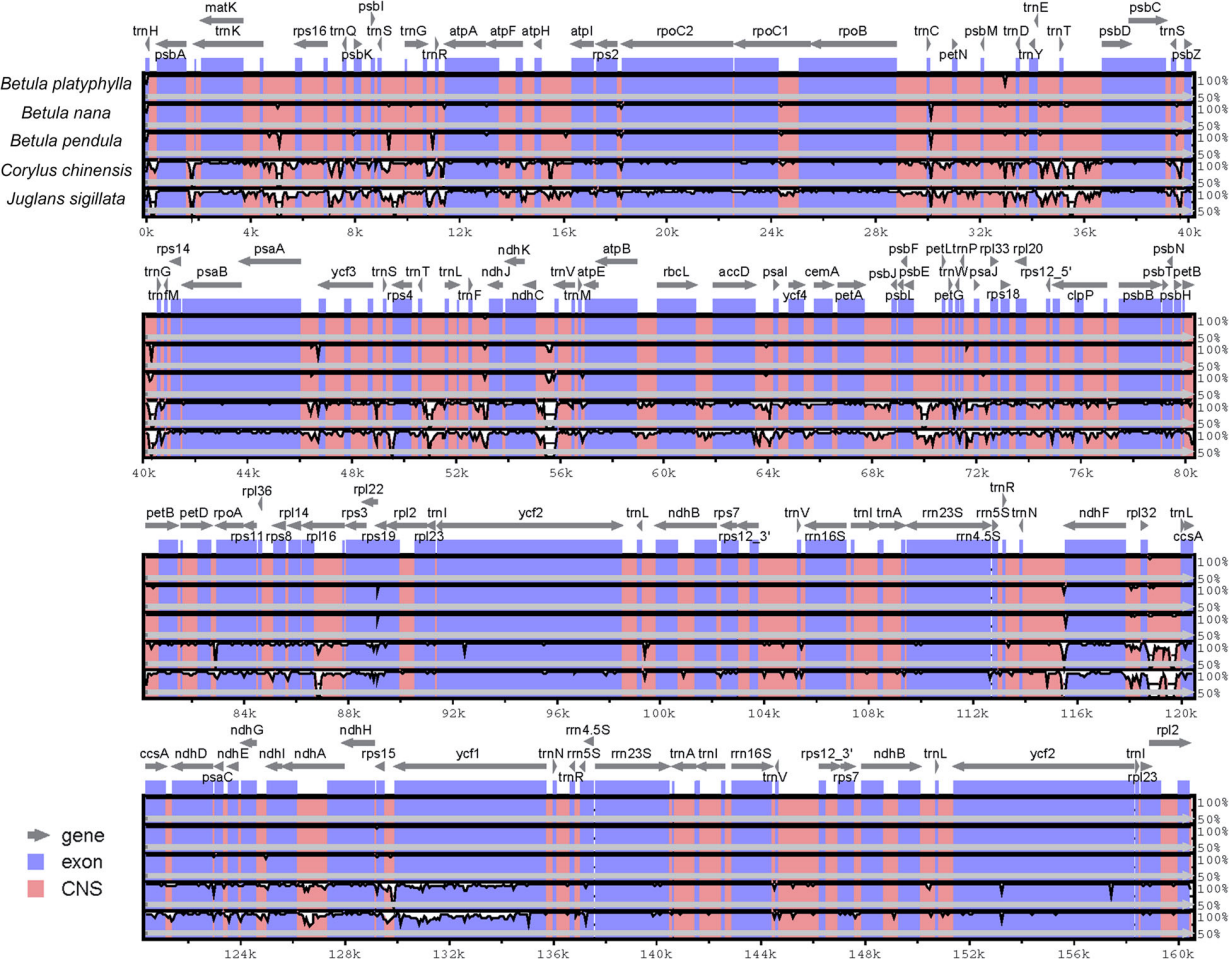
Sequence alignment of 5 chloroplast genomes in Fagales using the mVISTA program with *B. platyphylla* as a reference. Grey arrows above the alignment indicate the transcriptional directions of genes. Genome regions are color-coded as exon and conserved non-coding sequences (CNS). A cut-off of 50% identity was used for the plots. The Y-axis indicates the percent identity between 50 and 100%

To investigate the similarities and differences of the cp genome sequences between *B. platyphylla* and other species of Fagales, a global alignment program was used to align these sequences. The result was plotted using the mVISTA tools with *B. platyphylla* as a reference (Fig. 3). Overall, these closely related species had little difference in cp genome size, ranging from 160,320 bp to 161,148 bp. The global patterns of sequence similarities among these sequences were very high, especially among the Betulaceae species, with over 99% identity. As shown in Fig. 3, the structures of these cp genomes were conserved, and neither translocations nor inversions were detected in the sequences. As expected, coding regions were revealed to be more conserved than noncoding regions. More concretely, most high polymorphic regions were located in the intergenic regions (such as *trnR-TCT*—*atpA*, *trnE-TTC*—*trnT-GGT*, *psbE*—*petL*, *rpl32*—*trnL-TAG*), but the *ycf1* gene had higher variability regions. These regions may be undergoing more rapid nucleotide substitution at the species level, which indicates the potential application of molecular markers for phylogenetic analyses and plant identification in Fagales.

### IR expansion and contraction

**Fig. 4:**
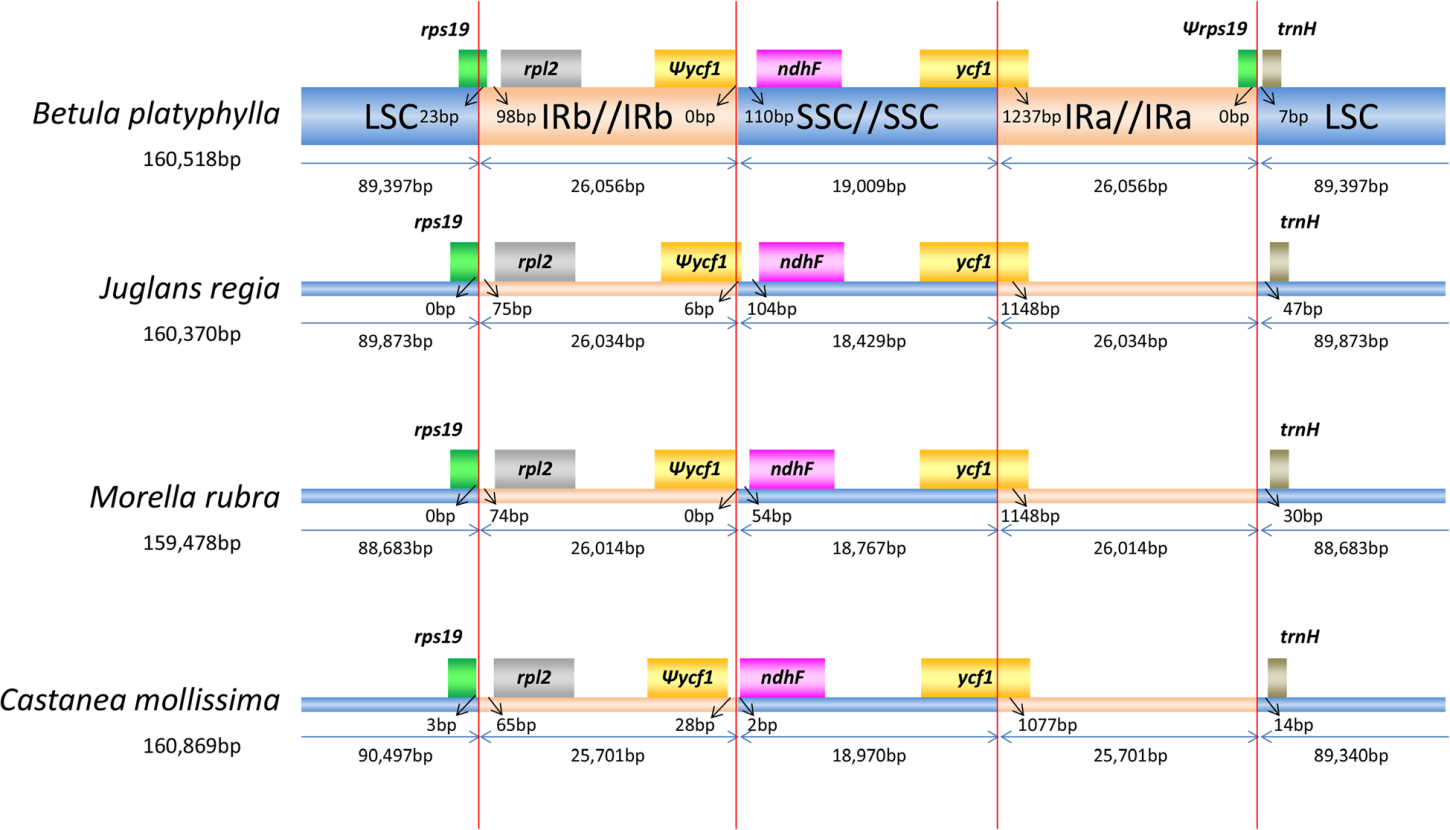
Comparison of the borders of LSC, SSC and IR regions among the four Fagales genomes. ψ indicates a pseudogene. The figure is not strictly proportional. Gene length: *B. platyphylla* (*rps19*: 279 bp; *rpl2*: 1511 bp, *ψycf1*: 1237 bp, *ndhF*: 2247 bp, *ycf1*: 5751 bp, *ψrps19*: 23 bp); *J. regia* (*rps19*: 285 bp, *rpl2*: 1510 bp, *ψycf1*: 1155 bp, *ndhF*: 2226 bp, *ycf1*: 5676 bp); *M. rubra* (*rps19*: 279 bp, *rpl2*: 1509 bp, *ψycf1*: 1149 bp, *ndhF*: 2226 bp, *ycf1*: 5655 bp); *C. mollissima* (*rps19*: 285 bp, *rpl2*: 1509 bp, *ψycf1*: 1050 bp, *ndhF*: 2262 bp, *ycf1*: 5685 bp)

IR expansions and contractions are common in cp genomes, which results in the variation in cp genome size. The differences in IRs may also reflect phylogenetic history. Here, we selected four representative species of the four families in Fagales and compared their sizes and the junctions of their LSC, SSC and IR regions. Although the lengths of the IR regions, ranging from 25,701 bp to 26,056 bp, varied little among the four species, some differences in the IR expansions and contractions were observed. As shown in Fig. 4, *B. platyphylla* did not show the longest total cp genome length among the four species, but its IR regions were the longest. The *rps19* genes of *J. regia*, *M. rubra* and *C. mollissima* were located in the LSC region, but the IRb region was expanded to include the *rps19* gene in *B. platyphylla*. Although the *rpl2* genes of the four species were all located completely within the IRb regions, the gene in *B. platyphylla* was at the farthest from the left boundary of the IR region. The *ψycf1* pseudogene was present in all four genomes, except in *J. regia*, in which it extends into the SSC region by several bases; the others were all located in the IRb regions. The distance between the *ψycf1* pseudogene and the *ndhF* gene decreased from *B. platyphylla* to *C. mollissima*. The IRa region extended into the *ycf1* gene in all these genomes, and the longest overlap between the IRa and *ycf1* genes was also observed in *B. platyphylla*. Of particular interest, the *ψrps19* pseudogene only existed in the *B. platyphylla* genome, which is located along the right boundary of the IRa region. The *trnH-GTG* genes were all located in the LSC region, at 7–47 bp apart from the IRa-LSC boundary. In summary, we found that the IR regions of the *B. platyphylla* cp genome were slightly expanded compared with that of the other three species.

### SSR analysis

Recently, an increasing number of theoretical reasons and well-documented examples show that the repetitive structure of genomic DNA is essential. It not only plays a major architectonic role in higher-order physical structuring but is also very useful in genome evolution and rearrangement [30, 31]. Here, we only focused on microsatellite sequences. A total of 105 SSRs were detected in the *B. platyphylla* cp genome (Additional file 1: Table S5). Among these SSRs, there were 53, 19, 15, 13 and 5 for mono-, di-, tri-, tetra- and penta- nucleotide repeats, respectively. No hexanucleotide repeats were found. A majority of the mononucleotides (98.1%) were composed of A/T, and most of the dinucleotides (84.2%) were AT (Additional file 1: Future S4). Because repeat sequences may be useful in developing lineage-specific markers, CandiSSR was used to identify candidate polymorphic SSRs among the cp genomes. Nevertheless, we only found one possible SSR locus among the three *Betula* species (Additional file 1: Table S6).

### Recognition of RNA editing sites

**Fig. 5:**
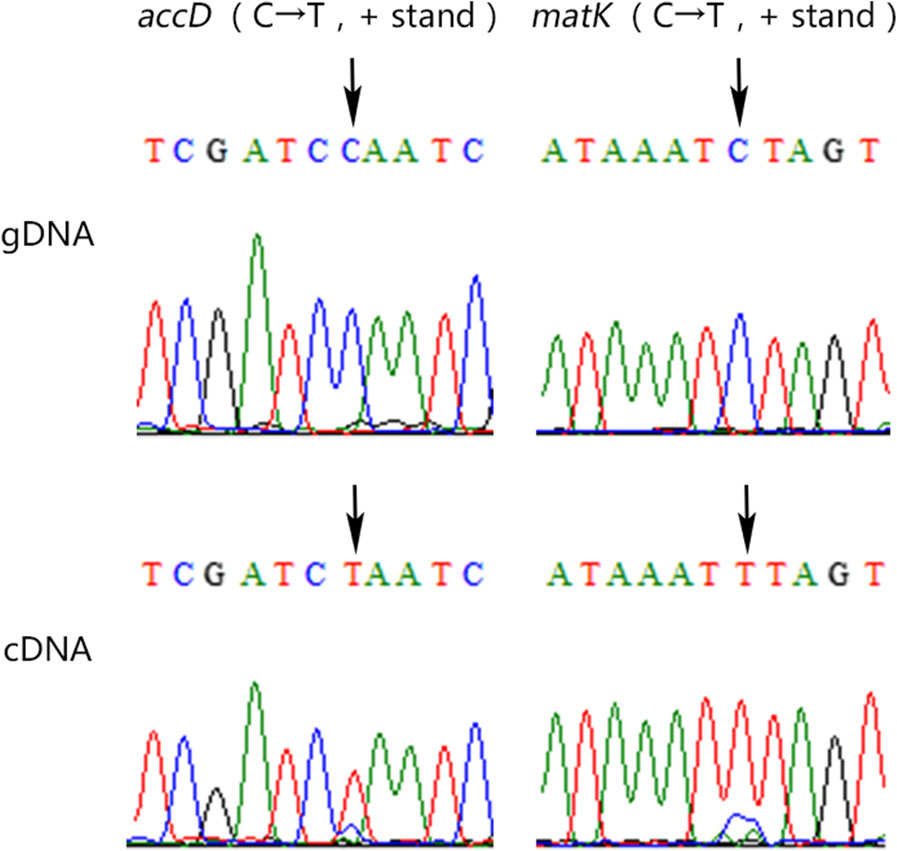
Validation of inferred editing sites from RNA-Seq by Sanger sequencing. Sequencing chromatogram traces from two exemplary gene loci, *accD* and *matK*, are shown. The editing positions are highlighted by arrows. The top trace is genomic DNA (gDNA), and the bottom trace is complementary DNA (cDNA)

To exhaustively analyze the *B. platyphylla* cp genome RNA editome, high-quality reads are necessary. After data cleaning, approximately 11.7 Gbp clean reads were obtained. The read alignment rates of the three biological repetitions to the reference *B. platyphylla* cp genome were only 1.23, 0.67 and 0.67%. However, a 95.3% region of the cp genome was covered with reads, and the average sequencing depth was over 650x. Furthermore, the distribution of reads was not uniform; the region in which tRNA and rRNA genes clustered in the IR had extremely high sequencing depth. The genome and gene body coverage maps are shown in the Additional file 1: Figures S2 and S3. Finally, we identified 80 RNA editing events in the whole *B. platyphylla* cp genome, of which 73 and 7 were located in gene and intergenic regions, respectively (Table 3). Among all of the edited genes, most of them were protein-coding genes, while 3 of them were rRNA genes. We did not discover any editing events that occurred in the tRNA transcripts. In protein-coding transcripts, editing events mainly occurred in the codon region, with only 1 in the 5’ UTR and 4 in introns. The largest number of editing sites was recognized in the *ndhB* gene (10 editing sites). A majority of changes in protein-coding transcripts converted C to U (5′-3′ direction of transcription), along with G to A and C to G in *rpoC2*, A to G in *ycf2* and G to A in *ndhF*. Among the edited codons, there were 9 (13.8%) changes at the first position, 49 (75.4%) at the second position and 7 (10.8%) at the third position. Most editing events (58 sites, 89.2%) lead to amino acid changes and editing in 7 loci (10.8%) did not alter amino acids (including stop codons). Among the 58 amino acid change sites, the largest proportion were Ser to Leu (25 sites, 43.1%), followed by Pro to Leu (12 sites, 20.7%) and Ser to Phe (9 sites, 15.5%). After comparison with the codon usage frequency and RSCU value at the editing sites, except for stop codon conversion, we found that all 6 other amino acid synonymous conversions increased the RSCU value of the codons, whereas the other nonsynonymous conversions did not show the same trend. For several editing sites located in the Intergenic region, nucleotide changes, including G to A, C to U and A to C (genome sequence direction as a positive chain), were observed. However, the editing sites located in the rRNA were the most specific; each site may be converted to more than two other bases. We also recognized that different editing sites had different editing efficiencies, from ~ 100% greater to ~ 10% poorer. As shown in Fig. 5, the C to T transitions at the 1406th base of the *accD* gene and at the 155th base of the *matK* gene were noticeable. After careful observation of the cDNA region in Fig. 5, it is not difficult to see that there is a weak cytosine (C) base signal under the peak of the corresponding editing site.

**Table 3:** RNA editing sites and amino acid changes

Gene	Subunit	Genome Position	Gene Position	Nucleotide Change	Codon Change	Edit Position within Codon	Amino Acid Change	Sequencing Depth	Editing Efficiency
*matK*	exon	2963	710	G > A	UCU > UUU	2	S > F	37	70
*matK*	exon	3518	155	G > A	UCU > UUU	2	S > F	38	84
*rps16*	exon	5795	212	G > A	UCA > UUA	2	S > L	179	82
*rps16*	intron	6389	–	G > A	–	–	–	47	81
*atpA*	exon	12,099	914	G > A	UCA > UUA	2	S > L	120	98
*atpA*	exon	12,222	791	G > A	CCG > CUG	2	P > L	189	98
*atpF*	exon	14,294	92	G > A	CCA > CUA	2	P > L	199	81
*IGR-1*	IGR	16,247	–	G > A	–	–	–	229	97
*rps2*	exon	17,781	248	G > A	UCA > UUA	2	S > L	85	95
*rpoC2*	exon	18,698	3761	G > A	UCA > UUA	2	S > L	41	98
*rpoC2*	exon	20,661	1798	G > T	GGU > AGU	1	G > S	33	18
*rpoC2*	exon	20,763	1696	G > C	CUC > GUC	1	L > V	34	12
*rpoC1*	exon	24,185	488	G > A	UCA > UUA	2	S > L	79	80
*rpoC1*	exon	25,453	41	G > A	UCA > UUA	2	S > L	20	65
*rpoB*	exon	26,307	2426	G > A	UCA > UUA	2	S > L	11	100
*rpoB*	exon	26,733	2000	G > A	UCU > UUU	2	S > F	15	93
*rpoB*	exon	27,555	1178	G > A	UCG > UUG	2	S > L	12	67
*rpoB*	exon	28,167	566	G > A	UCG > UUG	2	S > L	6	17
*rpoB*	exon	28,182	551	G > A	UCA > UUA	2	S > L	9	44
*rpoB*	exon	28,395	338	G > A	UCU > UUU	2	S > F	22	86
*IGR − 2*	IGR	35,186	–	A > C	–	–	–	23	17
*psbZ*	exon	39,947	50	C > U	UCA > UUA	2	S > L	176	26
*rps14*	exon	41,250	149	G > A	CCA > CUA	2	P > L	210	90
*rps14*	exon	41,319	80	G > A	CCC > CUC	2	P > L	240	88
*psaA*	exon	44,617	1395	G > A	CCC > CCU	3	P > P	95	37
*IGR-3*	IGR	51,329	–	C > U	–	–	–	614	17
*ndhK*	exon	54,554	65	G > A	UCA > UUA	2	S > L	826	89
*ndhC*	exon	54,719	323	G > A	ACU > AUU	2	T > I	816	96
*IGR-4*	IGR	59,062	–	G > A	–	–	–	349	28
*accD*	exon	62,731	815	C > U	UCG > UUG	2	S > L	65	92
*accD*	exon	63,322	1406	C > U	CCA > CUA	2	P > L	302	82
*psaI*	exon	64,351	88	C > U	CAU > UAU	1	H > Y	89	84
*cemA*	exon	66,260	492	C > U	UUC > UUU	3	F > F	84	49
*IGR-5*	IGR	67,928	–	C > U	–	–	–	31	13
*psbF*	exon	69,182	77	G > A	UCU > UUU	2	S > F	26	96
*psbF*	5’UTR	69,260	-2	G > A	–	–	–	39	62
*psbE*	exon	69,306	214	G > A	CCU > UCU	1	P > S	46	96
*petL*	exon	70,671	5	C > U	CCU > CUU	2	P > L	28	46
*IGR-6*	IGR	71,784	–	C > U	–	–	–	1163	99
*clpP*	exon	74,986	559	G > A	CAU > UAU	1	H > Y	33	97
*psbN*	exon	79,461	29	G > A	UCU > UUU	2	S > F	70	30
*petB*	intron	80,119	–	C > T	–	–	–	56	18
*petB*	exon	81,188	418	C > U	CGG > UGG	1	R > W	206	94
*petB*	exon	81,381	611	C > U	CCA > CUA	2	P > L	207	94
*rpoA*	exon	83,130	830	G > A	UCA > UUA	2	S > L	144	70
*rpoA*	exon	83,760	200	G > A	UCU > UUU	2	S > F	76	83
*rps11*	exon	84,338	108	G > A	UUC > UUU	3	F > F	357	55
*rpl23*	exon	91,217	89	G > A	UCA > UUA	2	S > L	25	84
*rpl23*	exon	91,235	71	G > A	UCU > UUU	2	S > F	31	97
*ycf2*	exon	98,495	6863	A > G	UAA > UGA	2	*>*	6	50
*ndhB*	exon	99,901	1487	G > A	CCA > CUA	2	P > L	20	80
*ndhB*	exon	99,955	1433	G > A	UCA > UUA	2	S > L	15	40
*ndhB*	exon	100,133	1255	G > A	CAU > UAU	1	H > Y	17	100
*ndhB*	exon	100,276	1112	G > A	UCA > UUA	2	S > L	25	88
*ndhB*	exon	100,558	830	G > A	UCA > UUA	2	S > L	10	70
*ndhB*	exon	101,328	746	G > A	UCU > UUU	2	S > F	9	22
*ndhB*	exon	101,337	737	G > A	CCA > CUA	2	P > L	11	64
*ndhB*	exon	101,488	586	G > A	CAU > UAU	1	H > Y	11	82
*ndhB*	exon	101,607	467	G > A	CCA > CUA	2	P > L	23	91
*ndhB*	exon	101,925	149	G > A	UCA > UUA	2	S > L	23	91
*rps12*	intron	103,149	–	G > A	–	–	–	87	67
*rps12*	intron	103,290	–	G > A	–	–	–	115	85
*IGR-7*	IGR	104,462	–	G > A	–	–	–	629	30
*rrn16S*	exon	106,489	577	G > A/U/C	–	–	–	1404	49
*rrn16S*	exon	107,021	45	U > A/G	–	–	–	1112	41
*rrn23S*	exon	111,418	881	U > A/G/C	–	–	–	3163	73
*ndhF*	exon	116,076	1734	C > U	AUG > AUA	3	M > I	23	26
*ndhF*	exon	117,520	290	G > A	UCA > UUA	2	S > L	21	38
*ndhD*	exon	121,618	1298	G > A	UCA > UUA	2	S > L	52	92
*ndhD*	exon	122,029	887	G > A	CCC > CUC	2	P > L	12	58
*ndhD*	exon	122,242	674	G > A	UCA > UUA	2	S > L	40	85
*ndhD*	exon	122,317	599	G > A	UCA > UUA	2	S > L	46	76
*ndhD*	exon	122,533	383	G > A	UCA > UUA	2	S > L	28	75
*ndhD*	exon	122,914	2	G > A	ACG > AUG	2	T > M	33	64
*ndhE*	exon	123,626	233	G > A	CCA > CUA	2	P > L	110	9
*ndhA*	exon	125,765	961	G > A	CCU > UCU	1	P > S	334	94
*ndhA*	exon	127,589	341	G > A	UCA > UUA	2	S > L	99	79
*ndhH*	exon	128,810	303	G > A	AUC > AUU	3	I > I	44	25
*ycf1*	exon	131,464	4236	G > A	CGC > CGU	3	R > R	160	19
*ycf1*	exon	133,945	1755	G > A	UUC > UUU	3	F > F	250	17

Editing efficiency is counted by edited reads divided by total mapped reads at the same site

If the sequencing depth of an editing site is less than 30, the edit rate may have a great error

The editing sites in the IR region are calculated only once

*IGR* Intergenic region

*UTR* untranslated region, it belongs to exon

*: Stop codon

### Phylogeny inference

**Fig. 6:**
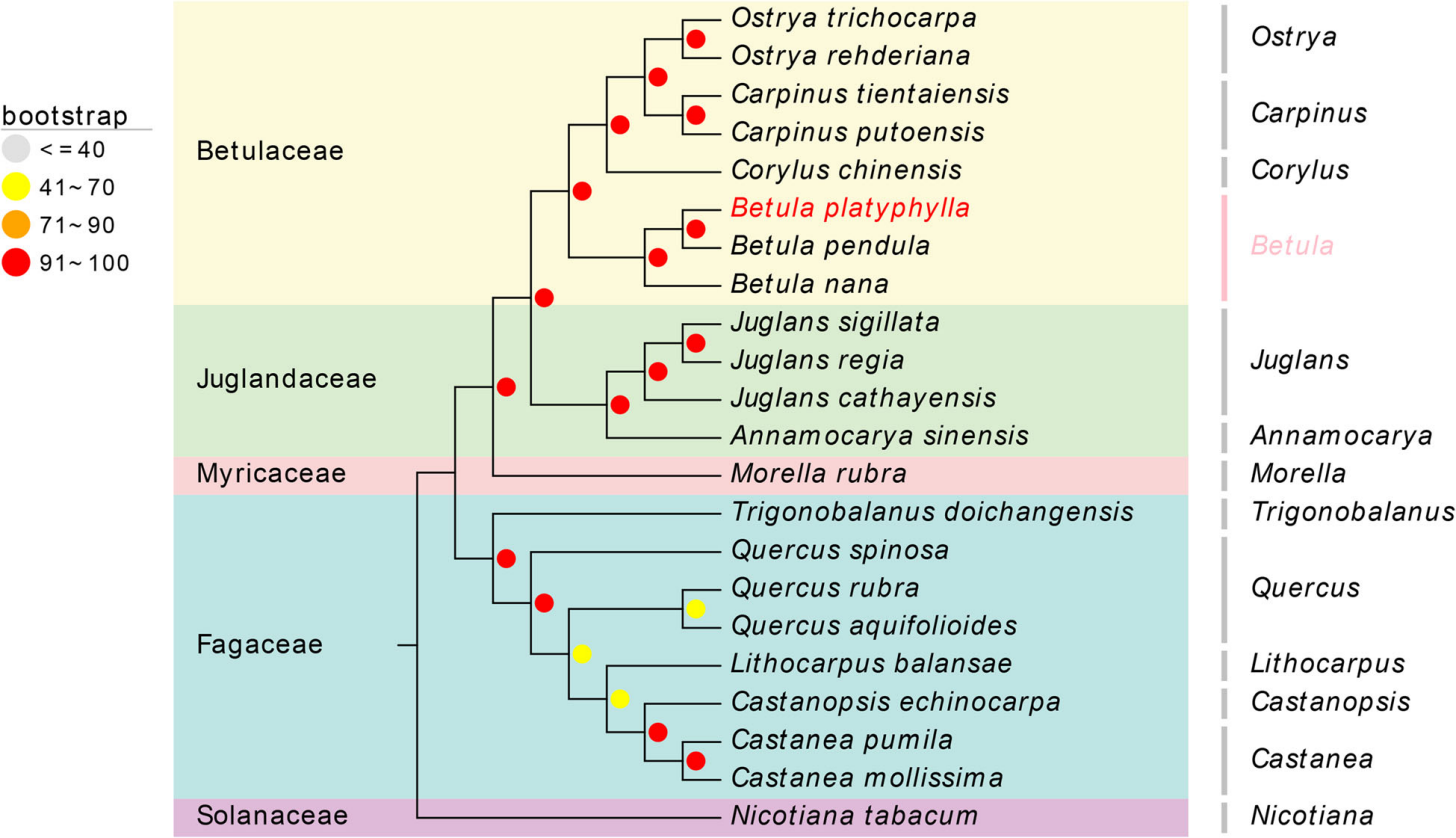
Phylogenetic tree reconstruction of Fagales using maximum likelihood (ML) based on whole chloroplast genome sequences. The ML bootstrap support value was given at each node. *Nicotiana tabacum* was used as the out-group

To determine the position of *B. platyphylla* in Fagales and to further analyze the relationships within Fagales, the complete cp genome sequences of 21 Fagales were obtained from the NCBI. Due to the high similarity of protein sequences in related species, we employed the whole cp genome sequences to build the phylogenetic tree to obtain more information. Model testing showed that GTR + G + I (General Time Reversible model, Gamma Distributed with Invariant Sites) was the optimal mode for whole-genome nucleotide sequences. In the latest APG IV system, Fagales includes seven families (Nothofagaceae, Fagaceae, Myricaceae, Juglandaceae, Ticodendraceae, Betulaceae, Casuarinaceae). In our study, the selected species only came from four families. Figure 6 clearly shows that all species formed four major clades, which correspond to the four selected families. In our ML tree, Fagaceae were sister to the remaining Fagales, followed by Myricaceae, which were subsequently sister to the remaining Fagales. Between *B. platyphylla*, *B. pendula* and *B. nana* of the genus *Betula* and family Betulaceae, *B. platyphylla* and *B. pendula* were located in adjacent branches, which means that they may have a closer evolutionary relationship.

## Discussion

The study presented here reported a complete cp genome sequence of *B. platyphylla*. In general, it had typical characteristics of angiosperm cp genomes and had little difference between closely related species. However, some of the phenomena attracted our attention.

Chloroplast microsatellites are some of the most common cp molecular markers. They are often used to investigate the evolution and systematics of genera and higher taxonomic ranks [32–36]. Although SSRs have now almost become the standard analysis in cp genome research, their scope of application is still open to question. Bang et al. have warned that attention must be paid to the risk of using amplicon-sized cpSSR markers for genetic relationship studies [37]. In our study, we found that there were more than 100 cpSSR loci in the *B. platyphylla* cp genome, but only one possible cpSSR locus among the three *Betula* species was predicted by CandiSSR. We also further identified the candidate polymorphic cpSSR loci among four typical species of Fagales (*B. platyphylla*, *C. mollissima*, *J. regia* and *M. rubra*) and obtained three possible loci. This seems to indicate that the closer the relationship between the species, the fewer candidate cpSSR loci were found for differentiating the species. For species that are closely related, a small number of cpSSRs may not be able to distinguish them accurately, especially depending on length alone. Therefore, we sometimes need more information, such as SNPs or INDEL, to differentiate closely related species.

We also reconstructed the phylogenetic tree of Fagales. Our ML tree formed four major clades and was highly consistent with previous research [38, 39]. Among the three closely related *Betula* species, *B. nana* is significantly different from the other two species, not only in morphology but also in the growth environment. The *Betula* branch had a strongly supported topology and showed that *B. platyphylla* and *B. pendula* have a closer evolutionary relationship. A previously reconstructed phylogeny for *Betula* species that is based on whole-genome SNP information reached the same conclusion [40].

RNA editing in the *B. platyphylla* cp genome is also one of the concerns of our study. As an important type of posttranscriptional regulation, it was previously widely believed that there were 30~50 RNA editing sites in a large majority of cp genomes [41–44]. The substitution is always either from cytidine (C) to uridine (U) or, less commonly, uridine (U) to cytidine (C) [45, 46]. In the past, software was used to predict RNA editing sites, but the software accuracy was generally low, and it could not predict synonymous mutation sites. In recent years, the advent of NGS improved the sensitivity of RNA editing site identification. An increasing number of editing sites that are not only in coding regions but also in noncoding and intergenic regions have been identified [47–49]. To reduce the false-positive, at most, one mismatch was allowed in an aligned reads and no gap open or gap extension was allowed when mapping to the reference genome. This inevitably lost some information and might miss possible editing sites that are very close to each other. However, it clearly improved the accuracy of the recognition. Finally, we identified 80 editing sites in the whole cp genome, including coding regions, noncoding regions and intergenic regions. Most of the substitutions were C to U, but a small number of other types that have never been reported in flowering plants were also detected. Except for the rRNAs, the editing sites that are not C to U are either low in sequencing depth or low in editing efficiency. This means that these edited transcripts are lower in expression, which seems to explain why these types of substitutions have not previously been discovered. Although we did not find any editing sites in *B. platyphylla* tRNA, some specific editing sites were identified in rRNAs. Unlike ordinary RNA editing, the editing sites in *B. platyphylla* rRNA can be replaced with different kinds of bases. Until now, the phenomenon of rRNA editing has been found only in algae chloroplasts [50–52]. Although other *B. platyphylla* RNA-Seq data also led to the same conclusion, we still doubted whether these editing sites were species specific. Excitingly, from public RNA-Seq data for *Arabidopsis thaliana* (https://www.ncbi.nlm.nih.gov/sra/, SRR682085, SRR6852086), we found almost the same editing modes at the same loci in the cp rRNA of *A. thaliana*. This indicates that cp rRNA editing may be somewhat conserved across species. It should be noted that most of the rRNAs were removed in the process of library construction; in fact, the expression of cp rRNA is far higher than the value we detected. Perhaps there is some mechanism to promote the conversion from one base to several other bases in a huge number of transcripts. In recent years, great progress has been made in the molecular mechanism of RNA editing, especially for C-to-U conversion. Some pentatricopeptide repeat (PPR) proteins and other factors, such as the proteins CRR, RIP/MORF, ORRM and OZ, have been found to form an RNA editosome and to guide the hydrolytic deamination of a cytosine to a uracil base [53–57]. Obviously, these studies are insufficient to elucidate the essence of the problem, especially for those other than for C-to-U editing. For a long time, RNA editing was believed to be a mechanism at the posttranscriptional level, probably acting as buffer to less favored mutations in the genomic coding sequences [56]. However, with the discovery of more editing sites, RNA editing seems to have some other effects. Notably, we found that, other than for the stop codon conversion, the 6 amino acid synonymous conversions increased the RSCU value which suggests a plant codon optimization phenomenon to improve protein expression. Moreover, there is no theoretical explanation for the cp rRNA editing phenomenon. Because rRNA is the main component of the ribosome and catalyzes peptide bond formation, these editing sites may change the ribosome structure or influence protein synthesis. Although sometimes RNA editing is important, is it necessary for all of these editing sites? Especially for those sites with low expression or low editing efficiency, we suspected that some of them may be the results of accidental effects of editosomes and transcripts. Of course, all of these conjectures require further confirmation through in vitro and in vivo studies.

The cp genome has obvious prokaryotic characteristics in codon selection, especially in initiation codon selection [58]. Many kinds of initiation codons, including ACG, GTG and ATA (but not ATG), have been found in chloroplasts, which is conserved across closely related species [59]. Although the sequence logo clearly shows the trend of initiation codon selection in different species, most of the current cp genome annotations were based on software prediction, which may be different from the real situation. The use of alternative initiation codons leads to two choices in the stages of translation and transcription: one is to use the Non-ATG as the start codon directly, and the other is to edit it back to ATG by RNA editing. In the *B. platyphylla* cp, the initiation codons of the *ndhD* transcripts were obviously edited, but those of *rps19* and *psbC* were not. The Shine–Dalgarno Sequence seems to facilitate translation initiation from the GUG [58, 60]. Recent studies have also surprised us with the translation of some unedited transcripts, which raises the possibility that ACG in *ndhD* can be utilized as an initiator codon in chloroplasts [61, 62]. Furthermore, attention should be given to whether unedited transcripts have some functions.

## Conclusions

In conclusion, we sequenced and investigated the complete cp genome sequence of *B. platyphylla*. The cp genome of *B. platyphylla* has a typical land plant cp genome structure and is highly similar to other cp genome sequences of Fagales. There were 3 genes using alternative initiation codons. More RNA editing sites were detected than ever before, and some had never been reported. This also helped us to determine the phylogenetic relationships among some species of Fagales. Our research will facilitate genomic, genetic engineering and phylogenetic studies of this important species. In the future, we will focus on molecular mechanisms that are involved in transcriptional regulation and translational modification of the cp by using new technologies and methods. We hope that these studies will help develop new varieties with higher photosynthetic efficiency.

## Additional file

Additional file 1: **Table S1.** Taxa and ID of the selected species. **Table S2.** The related information of primers. **Table S3.** Taxa and ID of the selected Fagales. **Table S4.** Codon usage frequency and RSCU value of the *B. platyphylla* chloroplast genome. **Table S5.** Simple sequence repeats within the *Betula platyphylla* chloroplast genome. **Table S6.** Candidate polymorphic SSRs and primers. **Figure S1.** Amino acid composition of protein-coding gene in the *B. platyphylla* chloroplast genome. **Figure S2.** Genome coverage distribution curve of RNA-Seq.Window length: 100 nt; step size: 50 nt. **Figure S3.** Gene body coverage distribution curve of RNA-Seq.All genes and sequencing depth have been normalized. **Figure S4.** Number of classified SSR repeat types (considering sequence complementary). (DOCX 2105 kb)

## Abbreviations

APG: Angiosperm Phylogeny Group
CNS: Conserved non-coding sequences
cp: Chloroplast
CTAB: Cetyl trimethylammonium bromide
IGR: Intergenic region
IR: Inverted repeat
LSC: Large single copy
ML: Maximum likelihood
NCBI: National Center for Biotechnology Information
NGS: Next-generation-sequencing
PPR: Pentatricopeptide repeat
RSCU: Relative synonymous codon usage
SNP: Single-nucleotide polymorphism
SSC: Small single copy
SSR: Simple sequence repeat
UTR: Untranslated region
