# Supplementary material for: Complete chloroplast genome sequence of Betula platyphylla: gene organization, RNA editing, and comparative and phylogenetic analyses
Source: BMC Genomics. 2018 Dec 20;19:950. doi: 10.1186/s12864-018-5346-x (PMC6302522; doi:10.1186/s12864-018-5346-x)
Supplement: Supplementary file 1 — Table S1. Taxa and ID of the selected species. Table S2. The related information of primers. Table S3. Taxa and ID of the selected Fagales. Table S4. Codon usage frequency and RSCU value of the B. platyphylla chloroplast genome. Table S5. Simple sequence repeats within the Betula platyphylla chloroplast genome. Table S6. Candidate polymorphic SSRs and primers. Figure S1. Amino acid composition of protein-coding gene in the B. platyphylla chloroplast genome. Figure S2. Genome coverage distribution curve of RNA-Seq.Window length: 100 nt; step size: 50 nt. Figure S3. Gene body coverage distribution curve of RNA-Seq.All genes and sequencing depth have been normalized. Figure S4. Number of classified SSR repeat types (considering sequence complementary). (DOCX 2105 kb) [file 12864_2018_5346_MOESM1_ESM.docx]

Additional file

**Table S1** Taxa and ID of the selected species

| Species name | GenBank accession number | Order | Family |
| --- | --- | --- | --- |
| *Amborella trichopoda* | AJ506156.2 | Amborellales | Amborellaceae |
| *Arabidopsis thaliana* | AP000423.1 | Brassicales | Brassicaceae |
| *Averrhoa carambola* | KU569488.1 | Oxalidales | Oxalidaceae |
| *Buxus microphylla* | EF380351.1 | Buxales | Buxaceae |
| *Camellia sinensis* | KC143082.1 | Ericales | Theaceae |
| *Cucumis sativus* | DQ119058.1 | Cucurbitales | Cucurbitaceae |
| *Daucus carota* | DQ898156.1 | Apiales | Apiaceae |
| *Euonymus schensianus* | NC_036019.1 | Celastrales | Celastraceae |
| *Glycine soja* | NC_022868.1 | Fabales | Fabaceae |
| *Gossypium raimondii* | NC_016668.1 | Malvales | Malvaceae |
| *Helianthus annuus* | KU306406.1 | Asterales | Asteraceae |
| *Hippophae rhamnoides* | NC_035548.1 | Rosales | Elaeagnaceae |
| *Iris sanguinea* | NC_029227.1 | Asparagales | Iridaceae |
| *Malus doumeri* | KX499861.1 | Rosales | Rosaceae |
| *Morus notabilis* | NC_027110.1 | Rosales | Moraceae |
| *Nandina domestica* | NC_008336.1 | Ranunculales | Berberidaceae |
| *Nicotiana tabacum* | Z00044.2 | Solanales | Solanaceae |
| *Oryza sativa* | NC_031333.1 | Poales | Poaceae |
| *Paeonia obovate* | NC_026076.1 | Saxifragales | Paeoniaceae |
| *Panax ginseng* | AY582139.1 | Apiales | Araliaceae |
| *Platanus occidentalis* | NC_008335.1 | Proteales | Platanaceae |
| *Populus trichocarpa* | EF489041.1 | Malpighiales | Salicaceae |
| *Ranunculus macranthus* | NC_008796.1 | Ranunculales | Ranunculaceae |
| *Salix babylonica* | MF189167.1 | Malpighiales | Salicaceae |
| *Solanum lycopersicum* | NC_007898.3 | Solanales | Solanaceae |
| *Spinacia oleracea* | AJ400848.1 | Caryophyllales | Amaranthaceae |
| *Theobroma cacao* | HQ336404.2 | Malvales | Malvaceae |
| *Triticum aestivum* | KC912694.1 | Poales | Poaceae |
| *Vitis vinifera* | NC_007957.1 | Vitales | Vitaceae |
| *Zea mays* | X86563.2 | Poales | Poaceae |

All the classifications are based on the Angiosperm Phylogeny Group (APG) IV 2016.

**Table S2** The related information of primers

| Target gene | Product Length (bp) | Forward (F) primer sequence | Reverse (R) primer sequence | Product Tm F/R (℃) |
| --- | --- | --- | --- | --- |
| *accD* | 181 | TGCCTACATTGCATTTGCGGGTAAA | CAGAGGAAAGAAAGCATGGAGCTGAA | 62.1/61.6 |
| *matK* | 768 | AGGCAACATGACTTCCTATACCCACTT | CTTGAGGAAGCATAGGATGCCCTGAAA | 62.2/63.2 |

**Table S3** Taxa and ID of the selected Fagales

| Species name | GenBank accession number | Family | Genus |
| --- | --- | --- | --- |
| *Annamocarya sinensis* | KX703001.1 | Juglandaceae | *Annamocarya* |
| *Betula nana* | NC_033978.1 | Betulaceae | *Betula* |
| *Betula pendula* | LT855378.1 | Betulaceae | *Betula* |
| *Carpinus putoensis* | KX695124.1 | Betulaceae | *Carpinus* |
| *Carpinus tientaiensis* | NC_034910.1 | Betulaceae | *Carpinus* |
| *Castanea mollissima* | KY951992.1 | Fagaceae | *Castanea* |
| *Castanea pumila* | KM360048.1 | Fagaceae | *Castanea* |
| *Castanopsis echinocarpa* | NC_023801.1 | Fagaceae | *Castanopsis* |
| *Corylus chinensis* | KX814336.2 | Betulaceae | *Corylus* |
| *Juglans cathayensis* | NC_033893.1 | Juglandaceae | *Juglans* |
| *Juglans regia* | MF167463.1 | Juglandaceae | *Juglans* |
| *Juglans sigillata* | KX424843.1 | Juglandaceae | *Juglans* |
| *Lithocarpus balansae* | KP299291.1 | Fagaceae | *Lithocarpus* |
| *Morella rubra* | NC_035006.1 | Myricaceae | *Morella* |
| *Ostrya rehderiana* | NC_028349.1 | Betulaceae | *Ostrya* |
| *Ostrya trichocarpa* | NC_034295.1 | Betulaceae | *Ostrya* |
| *Quercus aquifolioides* | NC_026913.1 | Fagaceae | *Quercus* |
| *Quercus rubra* | JX970937.1 | Fagaceae | *Quercus* |
| *Quercus spinose* | NC_026907.1 | Fagaceae | *Quercus* |
| *Trigonobalanus doichangensis* | NC_023959.1 | Fagaceae | *Trigonobalanus* |

All the classifications are based on the Angiosperm Phylogeny Group (APG) IV 2016.

**Table S4** Codon usage frequency and RSCU value of the *B. platyphylla* chloroplast genome

| Codon | AA | Count | RSCU | Codon | AA | Count | RSCU |
| --- | --- | --- | --- | --- | --- | --- | --- |
| AAA | Lys | 1,054 | 1.48 | GAA | Glu | 1,027 | 1.49 |
| AAC | Asn | 308 | 0.47 | GAC | Asp | 213 | 0.39 |
| AAG | Lys | 367 | 0.52 | GAG | Glu | 352 | 0.51 |
| AAU | Asn | 1,007 | 1.53 | GAU | Asp | 866 | 1.61 |
| ACA | Thr | 395 | 1.21 | GCA | Ala | 394 | 1.14 |
| ACC | Thr | 227 | 0.7 | GCC | Ala | 210 | 0.61 |
| ACG | Thr | 143 | 0.44 | GCG | Ala | 155 | 0.45 |
| ACU | Thr | 537 | 1.65 | GCU | Ala | 627 | 1.81 |
| AGA | Arg | 491 | 1.87 | GGA | Gly | 723 | 1.63 |
| AGC | Ser | 116 | 0.35 | GGC | Gly | 179 | 0.4 |
| AGG | Arg | 173 | 0.66 | GGG | Gly | 277 | 0.63 |
| AGU | Ser | 397 | 1.21 | GGU | Gly | 592 | 1.34 |
| AUA | Ile | 769 | 0.98 | GUA | Val | 550 | 1.55 |
| AUC | Ile | 448 | 0.57 | GUC | Val | 176 | 0.5 |
| AUG | Met | 603 | 1 | GUG | Val | 185 | 0.52 |
| AUU | Ile | 1,142 | 1.45 | GUU | Val | 509 | 1.43 |
| CAA | Gln | 716 | 1.54 | UAA | * | 50 | 1.79 |
| CAC | His | 135 | 0.43 | UAC | Tyr | 203 | 0.41 |
| CAG | Gln | 212 | 0.46 | UAG | * | 19 | 0.68 |
| CAU | His | 487 | 1.57 | UAU | Tyr | 794 | 1.59 |
| CCA | Pro | 311 | 1.14 | UCA | Ser | 409 | 1.25 |
| CCC | Pro | 207 | 0.76 | UCC | Ser | 302 | 0.92 |
| CCG | Pro | 152 | 0.56 | UCG | Ser | 185 | 0.56 |
| CCU | Pro | 425 | 1.55 | UCU | Ser | 562 | 1.71 |
| CGA | Arg | 354 | 1.35 | UGA | * | 15 | 0.54 |
| CGC | Arg | 104 | 0.4 | UGC | Cys | 85 | 0.56 |
| CGG | Arg | 112 | 0.43 | UGG | Trp | 449 | 1 |
| CGU | Arg | 338 | 1.29 | UGU | Cys | 219 | 1.44 |
| CUA | Leu | 384 | 0.84 | UUA | Leu | 861 | 1.87 |
| CUC | Leu | 175 | 0.38 | UUC | Phe | 523 | 0.71 |
| CUG | Leu | 177 | 0.39 | UUG | Leu | 578 | 1.26 |
| CUU | Leu | 583 | 1.27 | UUU | Phe | 960 | 1.29 |

* is stop codon.

Here we used RNA sequences without editing to compute the codon usage and RSCU values.

**Table S5** Simple sequence repeats within the *Betula platyphylla* chloroplast genome

| No. | SSR type | Size | Region | Start | End |
| --- | --- | --- | --- | --- | --- |
| 1 | (A)10 | 10 | LSC | 210 | 219 |
| 2 | (TA)5 | 10 | LSC | 1,777 | 1,786 |
| 3 | (AAT)4 | 12 | LSC | 3,047 | 3,058 |
| 4 | (ATT)5 | 15 | LSC | 4,692 | 4,706 |
| 5-6 | (AT)5tttttatttctttattatttattatttctaatttatttagaaataataaataacacaaataagtta(T)11 | 87 | LSC | 7,050 | 7,136 |
| 7-8 | (A)14gagaaagttgtttaaaatt(A)11 | 44 | LSC | 7,399 | 7,442 |
| 9 | (A)16 | 16 | LSC | 7,922 | 7,937 |
| 10 | (A)10 | 10 | LSC | 8,271 | 8,280 |
| 11 | (T)11 | 11 | LSC | 8,599 | 8,609 |
| 12 | (TCTT)3 | 12 | LSC | 9,036 | 9,047 |
| 13 | (AT)5 | 10 | LSC | 9,247 | 9,256 |
| 14 | (T)10 | 10 | LSC | 9,475 | 9,484 |
| 15 | (A)10 | 10 | LSC | 9,638 | 9,647 |
| 16 | (A)11 | 11 | LSC | 10,129 | 10,139 |
| 17 | (TTA)4 | 12 | LSC | 10,782 | 10,793 |
| 18 | (AAT)4 | 12 | LSC | 10,926 | 10,937 |
| 19 | (AT)6 | 12 | LSC | 11,368 | 11,379 |
| 20-21 | (T)10c(A)11 | 22 | LSC | 13,029 | 13,050 |
| 22 | (T)10 | 10 | LSC | 13,745 | 13,754 |
| 23 | (TA)5 | 10 | LSC | 14,441 | 14,450 |
| 24 | (CTC)4 | 12 | LSC | 15,739 | 15,750 |
| 25 | (T)10 | 10 | LSC | 16,004 | 16,013 |
| 26-28 | (T)12aaagaggaggtaccctgaaat(ATAA)3ttgttccgatggaaccttcttttctacctctaacggatattagccgttcatacatgattcaagccattaatttct(TTCTA)3 | 135 | LSC | 18,051 | 18,185 |
| 29 | (T)10 | 10 | LSC | 20,176 | 20,185 |
| 30 | (T)11 | 11 | LSC | 20,304 | 20,314 |
| 31 | (T)12 | 12 | LSC | 20,842 | 20,853 |
| 32 | (AT)5 | 10 | LSC | 21,683 | 21,692 |
| 33-34 | (C)10(A)10 | 20 | LSC | 24,370 | 24,389 |
| 35 | (T)10 | 10 | LSC | 24,786 | 24,795 |
| 36 | (T)10 | 10 | LSC | 28,087 | 28,096 |
| 37 | (AATA)3 | 12 | LSC | 33,268 | 33,279 |
| 38 | (A)10 | 10 | LSC | 33,692 | 33,701 |
| 39 | (A)11 | 11 | LSC | 34,471 | 34,481 |
| 40 | (AT)5 | 10 | LSC | 35,027 | 35,036 |
| 41-42 | (TTAT)3atttatttatattatttatattatttttattttatattattataataatataattataatga(ATT)4 | 86 | LSC | 35,437 | 35,522 |
| 43-44 | (TCTA)3tctgtatatatac(AGAT)3 | 37 | LSC | 39,565 | 39,601 |
| 45 | (TTAAG)4 | 20 | LSC | 40,702 | 40,721 |
| 46 | (A)10 | 10 | LSC | 46,523 | 46,532 |
| 47-48 | (A)11t(A)10 | 22 | LSC | 48,883 | 48,904 |
| 49 | (T)11 | 11 | LSC | 49,064 | 49,074 |
| 50 | (A)10 | 10 | LSC | 51,496 | 51,505 |
| 51 | (T)10 | 10 | LSC | 52,386 | 52,395 |
| 52 | (A)10 | 10 | LSC | 52,746 | 52,755 |
| 53 | (T)12 | 12 | LSC | 54,632 | 54,643 |
| 54-57 | (TAT)4ta(ATT)4aattattaaatttattattaaa(TAT)5taaatat(TAA)4 | 82 | LSC | 55,519 | 55,600 |
| 58 | (AACAA)3 | 15 | LSC | 56,993 | 57,007 |
| 59 | (T)10 | 10 | LSC | 58,862 | 58,871 |
| 60 | (ATT)4 | 12 | LSC | 63,444 | 63,455 |
| 61 | (TA)5 | 10 | LSC | 64,035 | 64,044 |
| 62 | (T)10 | 10 | LSC | 65,373 | 65,382 |
| 63 | (TC)5 | 10 | LSC | 65,836 | 65,845 |
| 64-66 | (A)10tttttaattttgattaaatactaaaatacatcgagtaaggttcgattagtaaagaatttgcatgatatctgatcaattgatcaatagaa(AT)7tattggtattttgtgattgtgtcctccagaaaaaaagggggggtccagtga(TTCT)3 | 176 | LSC | 68,285 | 68,460 |
| 67 | (GAAA)3 | 12 | LSC | 69,930 | 69,941 |
| 68 | (T)10 | 10 | LSC | 72,488 | 72,497 |
| 69 | (TA)6 | 12 | LSC | 72,848 | 72,859 |
| 70 | (A)10 | 10 | LSC | 73,343 | 73,352 |
| 71 | (A)11 | 11 | LSC | 75,670 | 75,680 |
| 72 | (AT)8 | 16 | LSC | 76,304 | 76,319 |
| 73 | (A)11 | 11 | LSC | 77,131 | 77,141 |
| 74 | (T)10 | 10 | LSC | 82,884 | 82,893 |
| 75-76 | (T)10ctttctttatatataatatata(T)10 | 42 | LSC | 85,616 | 85,657 |
| 77 | (T)10 | 10 | LSC | 86,131 | 86,140 |
| 78 | (GCAAT)3 | 15 | IR | 104,676 | 104,690 |
| 79 | (AGGT)3 | 12 | IR | 111,143 | 111,154 |
| 80 | (AG)5 | 10 | IR | 113,033 | 113,042 |
| 81 | (ATT)4 | 12 | SSC | 116,295 | 116,306 |
| 82 | (AAAT)3 | 12 | SSC | 117,812 | 117,823 |
| 83 | (T)11 | 11 | SSC | 118,063 | 118,073 |
| 84 | (AAAT)3 | 12 | SSC | 118,614 | 118,625 |
| 85 | (TA)5 | 10 | SSC | 119,126 | 119,135 |
| 86 | (TA)5 | 10 | SSC | 119,282 | 119,291 |
| 87-88 | (T)11cc(T)14 | 27 | SSC | 119,683 | 119,709 |
| 89 | (TAT)4 | 12 | SSC | 121,359 | 121,370 |
| 90-91 | (A)10tagaatatattttttcgagcagggggatttttgtcggt(A)10 | 58 | SSC | 122,958 | 123,015 |
| 92 | (TTGA)3 | 12 | SSC | 123,570 | 123,581 |
| 93-94 | (TA)5tttttatatattttttttta(T)10 | 40 | SSC | 125,026 | 125,065 |
| 95 | (T)10 | 10 | SSC | 126,456 | 126,465 |
| 96-97 | (TCT)4gtttatttatgcgcaactctttaattcttcttatacatcgaagacgagactcaataattttactgcaa(AT)7 | 94 | SSC | 127,122 | 127,215 |
| 98-99 | (AT)5ttcttgatcag(TAA)5 | 36 | SSC | 129,536 | 129,571 |
| 100 | (T)10 | 10 | SSC | 130,829 | 130,838 |
| 101-102 | (T)10aattttaa(T)11 | 29 | SSC | 132,772 | 132,800 |
| 103 | (CT)5 | 10 | IR | 136,874 | 136,883 |
| 104 | (CTAC)3 | 12 | IR | 138,760 | 138,771 |
| 105 | (CATTG)3 | 15 | IR | 145,225 | 145,239 |

**Table S6** Candidate polymorphic SSRs and primers

| SSR No. | Forward primer | Tm(℃) | Reverse primer | Tm(℃) |
| --- | --- | --- | --- | --- |
| 54-57 | TGTCACGTTTCAAGATTCATCCAC | 57.7 | GAGAAGGTCTACGGTTCGAGTCC | 59.7 |

The SSR number is in accordance with Supplementary Table 5.


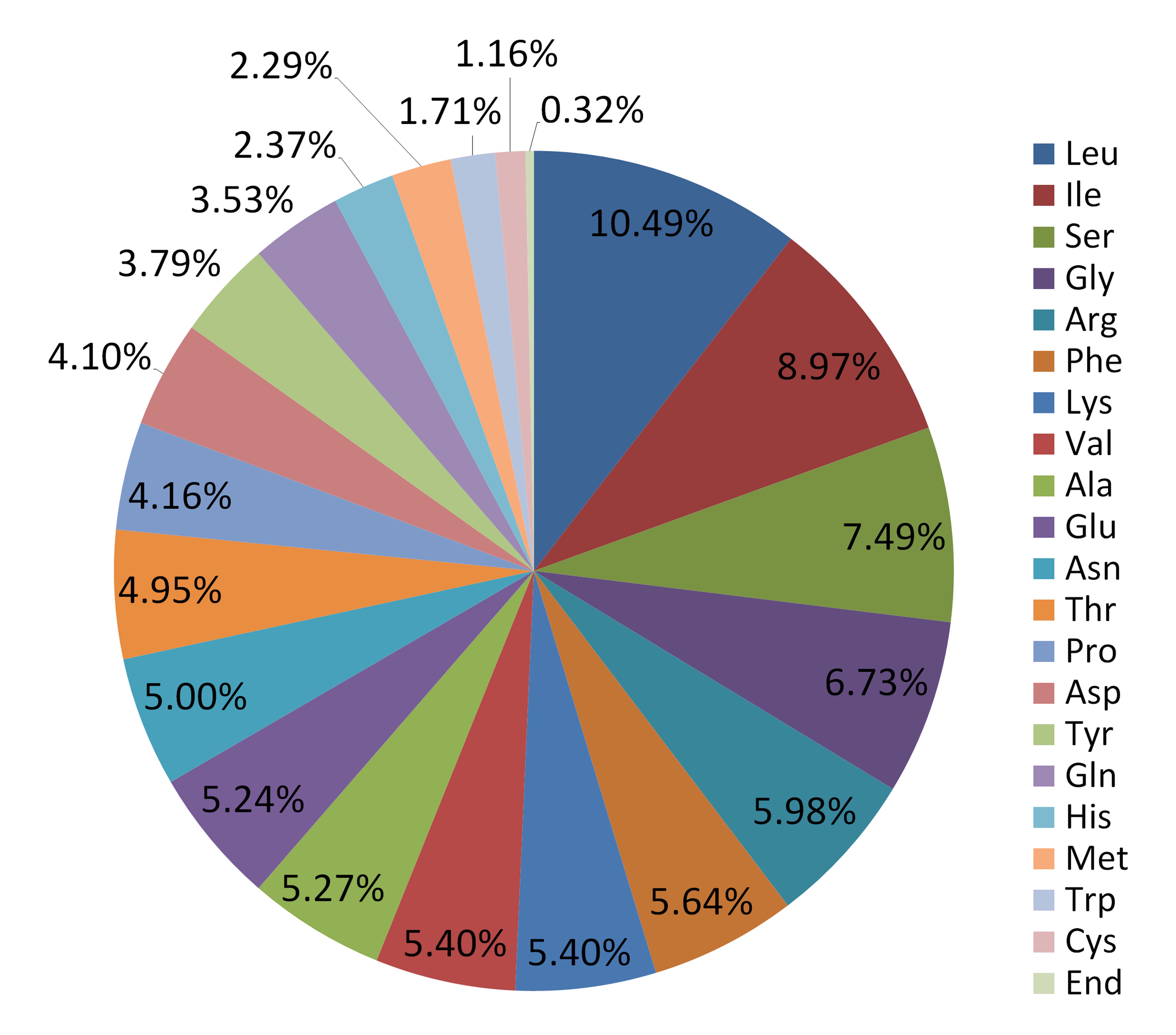


**Figure S1** Amino acid composition of protein-coding gene in the *B. platyphylla* chloroplast genome.





**Figure S2** Genome coverage distribution curve of RNA-Seq.

Window length: 100 nt; step size: 50 nt.


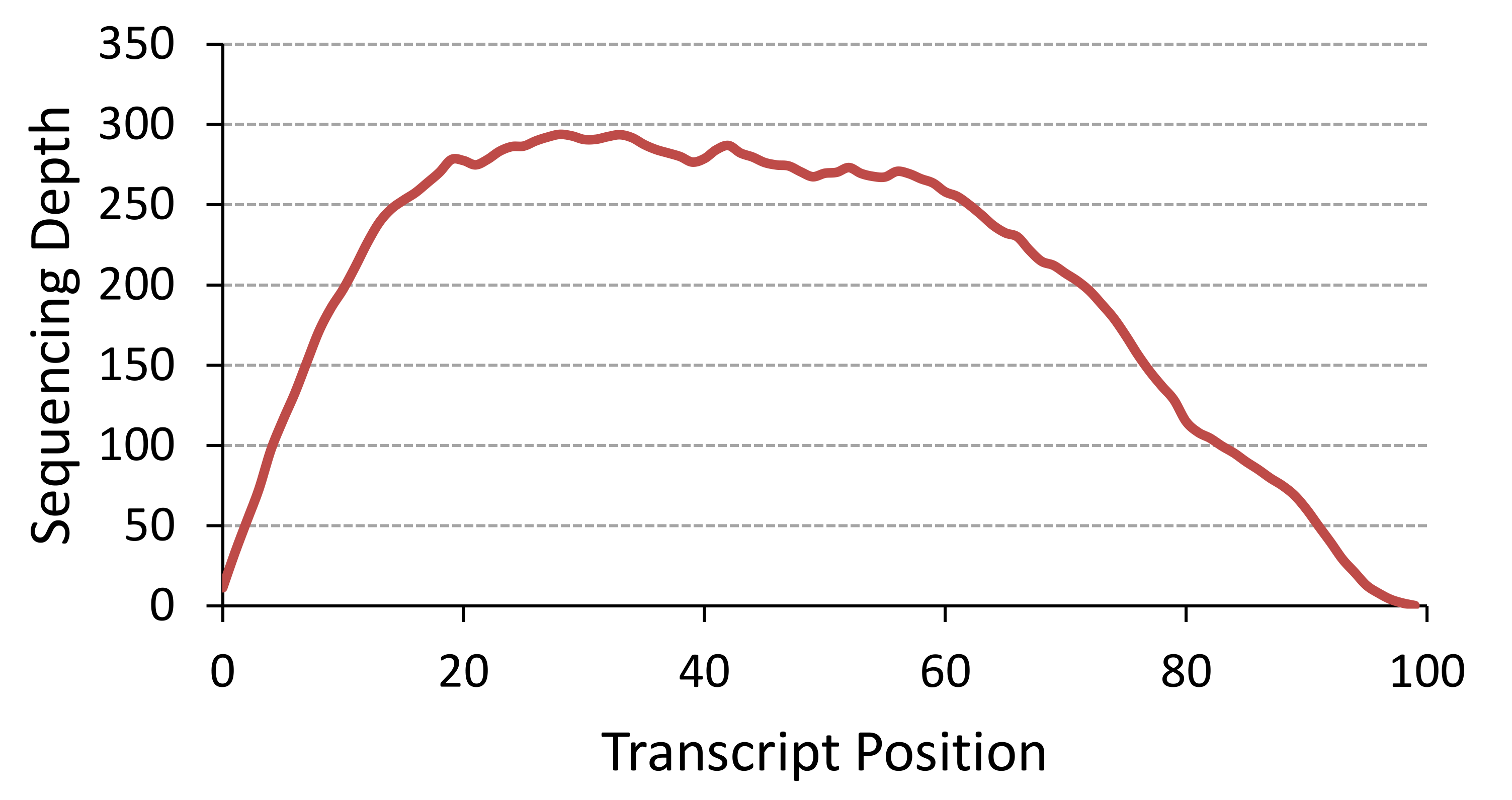


**Figure S3** Gene body coverage distribution curve of RNA-Seq.

All genes and sequencing depth have been normalized.


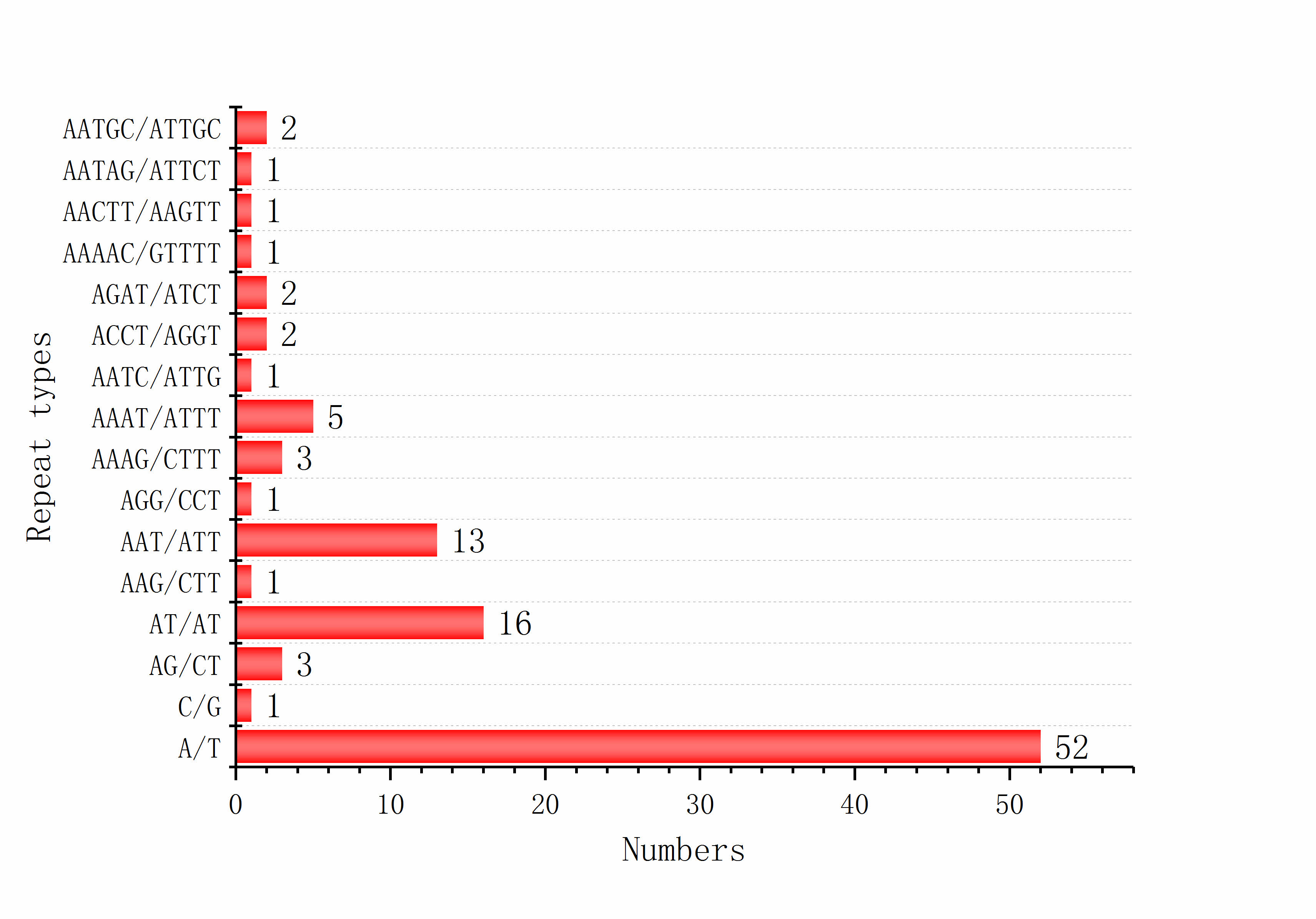


**Figure S4** Number of classified SSR repeat types (considering sequence complementary).

**Note S1**

All these 49 conserved chloroplast proteins are atpA, atpB, atpE, atpF, atpH, ndhB, ndhC, ndhE, ndhF, ndhH, ndhI, ndhJ, petA, petB, petD, petG, psaA, psaB, psaJ, psbA, psbC, psbD, psbE, psbF, psbH, psbJ, psbK, psbN, rpl16, rpl2, rpl20, rpl23, rpl33, rpoA, rpoB, rpoC1, rpoC2, rps11, rps12, rps14, rps15, rps19, rps2, rps3, rps4, rps7, rps8, ycf2, ycf3.
